# 37 Procedural Pain Management in Burn Patients (Phase II) - A Quality Improvement Project

**DOI:** 10.1093/jbcr/irac012.040

**Published:** 2022-03-23

**Authors:** Albert Pedroza, Zack Fleishhacker, Colette Galet, Lucy Wibbenmeyer

**Affiliations:** University of Iowa Carver College of Medicine, Iowa City, Iowa; University of Iowa Carver College of Medicine, Iowa City, Iowa; University of Iowa, Iowa City, Iowa; University of Iowa MD, FACS, Iowa City, Iowa

## Abstract

**Introduction:**

This quality improvement project (QI) focused on reducing procedural pain. Phase I of this project demonstrated a need to improve our patients' and nurses’ satisfaction with pain control management during hydrotherapy. In this phase (Phase II), we educated the nursing staff on 1) appropriate dosing and timing of opioid administration (Oral and IV) and 2) need for more frequent midazolam administration before hydrotherapy. We also assessed the safety and potential benefits of ketamine for procedural pain control.

**Methods:**

Patients undergoing wound cleaning procedures were surveyed for up to three hydrotherapy encounters. Nurses were educated on procedural pain control prior to this current phase. A convenience sample of patients underwent ketamine administration. Ketamine was administered per protocol starting at 0.3 or 0.4 mg/kg with intermittent boluses of 0.25 mg/kg as needed. Demographics, opioid (oral morphine equivalents, OME), midazolam, and ketamine doses and time of administration, and adverse events were collected. Patient pain scores (1-10) before and after hydrotherapy and patient and nurse satisfaction scores (1-10) after hydrotherapy were collected. Paired t-tests and one-way ANOVA were performed. P < 0.05 was considered significant.

**Results:**

During Phase II, 9 patients and 19 encounters were analyzed; of which ketamine was administered for 10 events. Compared to Phase I (28 Patients and 50 encounters), education of the nursing staff resulted in a significant improvement in giving midazolam prior to hydrotherapy (78.9% vs. 10%; p < 0.001) and opioid administration (27.4 OME vs. 20.2 OME, p = 0.043). Timing of opioid administration did not improve when compared to Phase I (Outside the window of efficacy: 42.1% vs. 43.8%, p = 0.903). Both patient (8.7 ± 2.4 vs. 7.7 ± 2.1; p = 0.011) and nurse (8.2 ± 2.1 vs. 7.3 ± 1.9; p = 0.021) satisfaction scores regarding pain control improved. None of the patients who received ketamine experienced adverse effects. Notably, the use of ketamine improved nurse satisfaction scores with patient pain control (9.2 ± 0.8 vs. 7.1 ± 2.6; p = 0.035). While patients on ketamine were more satisfied with pain control, significance was not reached (9.4 ± 1.1 vs. 7.9 ± 3.2; p = 0.315).

**Conclusions:**

Our data demonstrate that nurse education regarding medication administration was effective, but further education is needed to improve the timing of opioid administration. Ketamine offers additional benefits that deserve additional study.

